# Target-specific compound selectivity for multi-target drug discovery and repurposing

**DOI:** 10.3389/fphar.2022.1003480

**Published:** 2022-09-23

**Authors:** Tianduanyi Wang, Otto I. Pulkkinen, Tero Aittokallio

**Affiliations:** ^1^ Institute for Molecular Medicine Finland (FIMM), University of Helsinki, Helsinki, Finland; ^2^ Department of Computer Science, Aalto University, Espoo, Finland; ^3^ Helsinki Institute for Information Technology (HIIT), Department of Computer Science, University of Helsinki, Helsinki, Finland; ^4^ Department of Mathematics and Statistics and InFLAMES Research Flagship, University of Turku, Turku, Finland; ^5^ Institute for Cancer Research, Department of Cancer Genetics, Oslo University Hospital, Oslo, Norway; ^6^ Oslo Centre for Biostatistics and Epidemiology (OCBE), Faculty of Medicine, University of Oslo, Oslo, Norway

**Keywords:** drug selectivity, drug repurposing, drug discovery and development, kinase inhibition activity, polypharmacological effects

## Abstract

Most drug molecules modulate multiple target proteins, leading either to therapeutic effects or unwanted side effects. Such target promiscuity partly contributes to high attrition rates and leads to wasted costs and time in the current drug discovery process, and makes the assessment of compound selectivity an important factor in drug development and repurposing efforts. Traditionally, selectivity of a compound is characterized in terms of its target activity profile (wide or narrow), which can be quantified using various statistical and information theoretic metrics. Even though the existing selectivity metrics are widely used for characterizing the overall selectivity of a compound, they fall short in quantifying how selective the compound is against a particular target protein (e.g., disease target of interest). We therefore extended the concept of compound selectivity towards target-specific selectivity, defined as the potency of a compound to bind to the particular protein in comparison to the other potential targets. We decompose the target-specific selectivity into two components: 1) the compound’s potency against the target of interest (absolute potency), and 2) the compound’s potency against the other targets (relative potency). The maximally selective compound-target pairs are then identified as a solution of a bi-objective optimization problem that simultaneously optimizes these two potency metrics. In computational experiments carried out using large-scale kinase inhibitor dataset, which represents a wide range of polypharmacological activities, we show how the optimization-based selectivity scoring offers a systematic approach to finding both potent and selective compounds against given kinase targets. Compared to the existing selectivity metrics, we show how the target-specific selectivity provides additional insights into the target selectivity and promiscuity of multi-targeting kinase inhibitors. Even though the selectivity score is shown to be relatively robust against both missing bioactivity values and the dataset size, we further developed a permutation-based procedure to calculate empirical *p*-values to assess the statistical significance of the observed selectivity of a compound-target pair in the given bioactivity dataset. We present several case studies that show how the target-specific selectivity can distinguish between highly selective and broadly-active kinase inhibitors, hence facilitating the discovery or repurposing of multi-targeting drugs.

## 1 Introduction

Compound selectivity is a critical factor when developing new drugs or repurposing existing drugs for new uses (Bosc et al., 2017; Schipper et al., 2022). Binding affinity measurements of a compound across various target proteins enable systematic mapping of the target activity space and bioactivity spectrum of the compound. If a compound has a narrow target profile and activity spectrum, i.e., it binds effectively to a few specific targets, then the compound is considered as more selective than a compound with a wide activity spectrum and which binds to multiple targets with similar affinities. The overall selectivity of a compound can therefore be characterized in terms of how narrow or wide its bioactivity spectrum is. Compounds that potently bind to a single target protein are often easier to develop and optimize for clinical use. However, most of the currently used drugs have relatively broad polypharmacological profile, that is, their phenotypic responses are due to interactions with multiple protein targets at different degrees of binding affinity. For instance, kinases are promising therapeutic targets for various indications, including cancer, autoimmune diseases, inflammatory diseases, and cardiovascular diseases, but due to their structural similarity, it is rather challenging to develop highly selective kinase inhibitors (Davis et al., 2011). However, such polypharmacological effects of kinase inhibitors make them also potential candidates for drug repurposing, provided the compound has sufficient selectivity against the off-target proteins driving the disease progression.

A number of statistical and information theoretic metrics have been introduced to quantify compound selectivity. For example, the standard selectivity score calculates the number of targets bound by a compound above a given binding affinity threshold (Karaman et al., 2008). The Gini selectivity metric quantifies how widely the binding affinity measurements of a compound are spread across the target space (Graczyk, 2007; Ursu et al., 2020). More specifically, if there are only a few high binding affinities in the bioactivity spectrum, while the rest of the target activities remain weak, then the binding affinities are unevenly distributed, thus resulting in a high Gini coefficient, and the compound is considered selective. The selectivity entropy also estimates how the binding affinities of a compound distribute across the target space (Uitdehaag and Zaman, 2011). A high entropy indicates that the compound binds to many targets at comparable affinities, and is hence considered non-selective, while low entropy indicates a strong binding to only a few targets, thus making the compound selective (Uitdehaag et al., 2012). While the dissociation constant K_d_ is often used as an estimate of the binding affinity, the Partition index makes use of association constant K_a_ instead (Cheng et al., 2010). Partition index quantifies the compound selectivity by calculating the fraction of binding strength (as measured by K_a_) to a reference target in comparison to other targets. Recently, the KInhibition Selectivity Score (KISS) was designed for percentage inhibition target activity data, with user-defined on- and off-targets as prior information (Bello and Gujral, 2018). In KISS calculation, penalties are placed on off-target effects by empirical penalty functions, so that lower penalty and higher on-target effects indicate that the compound is selective.

**FIGURE 1:**
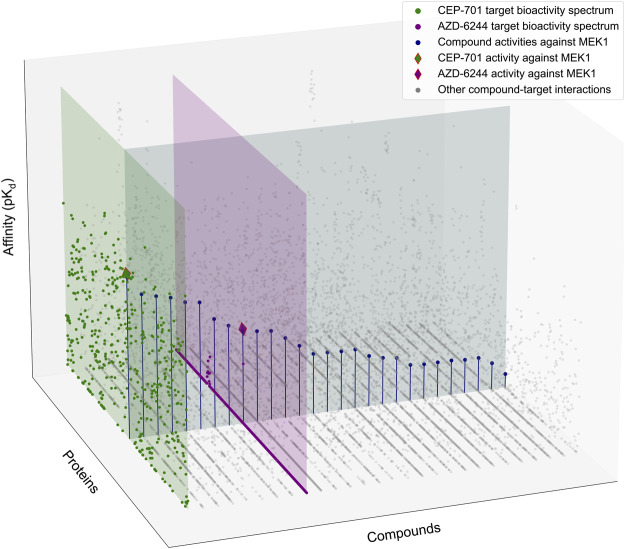
Schematic illustration of the target-specific drug selectivity concept. A subset of the Davis et al. dataset (Davis et al., 2011), where 28 randomly selected compounds and all 442 kinases were used for the illustration purposes. The gray horizontal panel shows the activity profile of the 28 kinase inhibitors against MEK1, where the compounds are ordered based on their relative potencies against MEK1. The green and purple vertical panels show the bioactivity spectrums of the compounds CEP-701 and AZD-6244, respectively, across the 442 kinase targets. Even though CEP-701 has the highest potency against MEK1 across all the compounds, it also has other high-potency targets, indicating that CEP-701 is not highly selective against MEK1. While AZD-6244 is not the most potent compound against MEK1, it has its highest potency against MEK1, and therefore AZD-6244 is considered as more selective against MEK1 than CEP-701.

The existing selectivity metrics estimate certain characteristics of a compound’s bioactivity spectrum from slightly different perspectives, hence leading to a variable performance in different drug discovery applications (Bosc et al., 2017; Miljković and Bajorath, 2018a; Miljković and Bajorath, 2018b). However, none of the existing metrics are designed for identifying selective compounds for a given target protein of interest. This is because the current selectivity metrics effectively estimate the narrowness of the bioactivity spectrum across the potential targets and consider a compound as highly selective if it binds to only a single target, regardless of the target identity. This makes it difficult to use these metrics for finding selective compounds against a specific target. A target-specific selectivity analysis is needed in many applications, e.g., when developing or repurposing drugs against a specific disease target, while guaranteeing that the drug should not have strong off-target activities toward other proteins which may lead to unwanted side effects (Aittokallio, 2022). To fill this gap, we introduce a target-specific compound selectivity scoring approach to facilitate identification of selective compounds against a given target protein (Figure 1). We demonstrate here the performance and use of the novel selectivity score in the context of kinase inhibitors, which are known to have a wide degree of polypharmacological activities, but the general approach is applicable also to other drug and target classes.

## 2 Results

### 2.1 Kinase target activity dataset for the selectivity scoring

**FIGURE 2:**
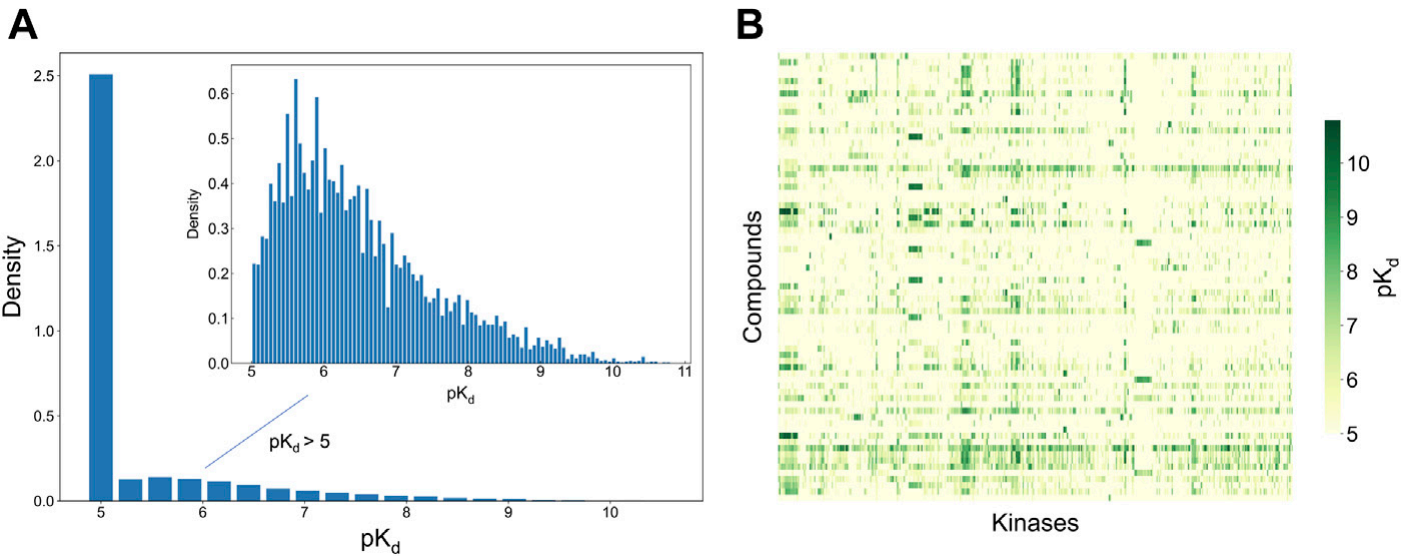
Bioactivity data (pK_d_ values) in the Davis dataset (Davis et al., 2011), containing 72 compounds and 442 kinases. **(A)** The bioactivity distributions, where the larger one includes all bioactivity data, and the smaller one (inset) includes only those bioactivities with pK_d_ > 5 (the pairs with K_d_ = 10 uM, i.e., pK_d_ = 5, indicate no activity in the primary screen). **(B)** Heatmap of the target activities. Higher pK_d_ (lower K_d_) values indicate stronger compound-kinase activities.

To develop and test the new selectivity score, we used a published dataset of fully-measured compound-target interactions between 72 kinase inhibitors and 442 kinases (Davis et al., 2011). Figure 2 shows the distribution of the measured compound-kinase interactions in terms of pK_d_. In this bioactivity data matrix, a large number of compound-kinase pairs show no activity, with pK_d_ = 5, i.e., K_d_ = 10 uM, and only a few compound-target pairs show strong potency, with pK_d_ > 9 i.e., K_d_ < 1 nM. As expected with kinase inhibitors that are known to have varied degrees of target promiscuity, many compounds have relatively strong activities against multiple kinases, and many kinases have a number of potent inhibitors. This makes the Davis et al. dataset an excellent test bench for developing and testing a new selectivity method, since it encompasses compounds and kinases with different polypharmacological activities and wide differences in their activity spectra, including both highly promiscuous compounds targeting multiple kinases at low concentrations, and highly selective compounds with narrow target activity profiles.

### 2.2 Decomposition of target-specific compound selectivity

Given a compound *c*_*i*_ ∈ *C* and a target *t*_*j*_ ∈ *T*, the bioactivity spectrum of the compound *c*_*i*_ can be defined as $B_{c_{i}}=\left\{K_{c_{i},\;t_{j}}\;|\;t_{j}\in T\right\}$ , and the activity profile of the target *t*_*j*_ can be defined as $P_{t_{j}}=\left\{K_{c_{i},\;t_{j}}\;|\;c_{i}\in C\right\}$ , where $K_{c_{i},\;t_{j}}$ is the interaction strength between *c*_*i*_ and *t*_*j*_ (here, dissociation constant K_d_, but in general it can be any binding affinity estimate).

The existing compound selectivity metrics try to characterize the distributional properties of $B_{c_{i}}$ , essentially measuring whether a compound interacts with only a few or larger number of targets. However, such a compound-specific approach is not sufficient when a specific protein target is under investigation. When assessing the target-specific compound selectivity, two aspects of the pairwise interactions need to be considered (1): how the interaction strength of a compound is distributed across its targets, i.e., characterizing $B_{c_{i}}$ ; and (2) how the interaction strength of a target is distributed across the compounds, i.e., characterizing $P_{t_{j}}$ (see the horizontal and vertical panels of Figure 1).

Given a set of compounds *C* and a set of targets *T*, that are explored in a target activity profiling study, the task of finding the most potent compounds and highest affinity targets among the compound and target spaces can be formulated as an optimization problem:

$$c*\left(t_{i}\right)=\underset{c_{i}}{argmax}P_{t_{j}},s.t.\;t_{j}\epsilon T$$

$$t*\left(c_{i}\right)=\underset{t_{i}}{argmax}B_{c_{j}},s.t.\;c_{i}\epsilon C$$

However, as was illustrated in Figure 1, the optimal solutions to these two objectives do not agree in general, i.e., the most potent compound c*(*t*_*j*_) (e.g., CEP-701 in Figure 1) for a target *t*_*j*_ (e.g., MEK1) is not necessarily among the compounds (e.g., AZD-6244) that each exert their highest affinity toward *t*_*j*_ and are considered selective in this respect. Likewise, the selective potency of a compound (AZD-6244) for a target (MEK1) does not imply that the most potent compound (CEP-701) for the same target shows superior potency over the other targets. Therefore, the target-specific selectivity needs to be formulated as a multi-objective optimization problem that considers both $B_{c_{i}}$ and $P_{t_{j}}$ .

Intuitively, for a target *t*_*j*_ *,* one tries to find the compound *c*_*i*_ that simultaneously maximizes $K_{c_{i},\;t_{j}}$ in $P_{t_{j}}$ and minimizes some statistic describing $B_{c_{i}}\backslash \left\{K_{c_{i},\;t_{j}}\right\}$ , for example, the mean of the set $B_{c_{i}}\backslash \left\{K_{c_{i},\;t_{j}}\right\}$ . In addition to the global mean, we also used a more local statistic by taking the mean of the *h*-nearest neighbors of $K_{c_{i},\;t_{j}}$ in $B_{c_{i}}$ , i.e., $B_{c_{i},\;hNN\left(t_{j}\right)}$ , where $hNN\left(t_{j}\right)$ denotes the *h*-nearest neighbors of $K_{c_{i},\;t_{j}}$ in $B_{c_{i}}$ in terms of target activity.

We formulated the above two statistics relative to $K_{c_{i},\;t_{j}}$ as below:

$${\mathrm{Global relative potency}\;G_{c_{i},\;t_{j}}=K}_{c_{i},\;t_{j}}-mean\left(B_{c_{i}}\backslash \left\{K_{c_{i},\;t_{j}}\right\}\right)$$ (1)

$$\mathrm{Local relative potency}\;{L_{c_{i},t_{j}}=K}_{c_{i},t_{j}}-mean\left(B_{c_{i},\;hNN\left(t_{j}\right)}\right)$$ (2)

Additionally, $K_{c_{i},\;t_{j}}$ is termed as *absolute potency*. Based on these definitions, the target-specific selectivity can be obtained as a solution of the bi-objective optimization problem, in which one maximizes simultaneously both the absolute potency and the relative potency, which can be easily solved using the ε-constraint method (Haimes, 1971; Miettinen, 1999) (see Materials and methods for details). Here, we used the neighborhood size of *h* = 5 in the local relative potency, unless otherwise specified.

**FIGURE 3:**
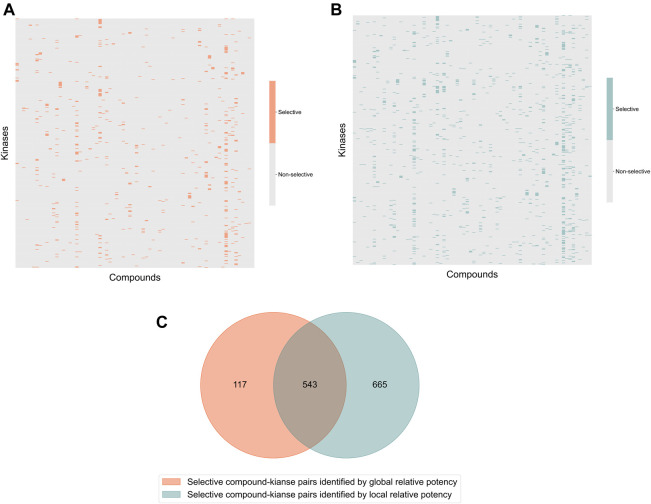
Heatmaps of the identified selective and broadly-active compound-kinase pairs among 72 compounds and 442 kinases when using **(A)** local relative potency and **(B)** global relative potency; **(C)** the overlap of the identified selective compound-kinase pairs identified using the local and global relative potency.

In the Davis dataset, 1,208 selective compound-kinase pairs were identified among the 31,824 total pairs between 72 compounds and 442 kinases when using the local relative potency (Figure 3A); while using the global relative potency, 660 selective pairs were identified (Figure 3B). Even if the use of the local relative potency in the optimization problem led to 1.8-fold more selective compound-target pairs, compared to using the global relative potency, there is still a relatively large overlap between the identified selective compound-target pairs (Figure 3C). Since the local and global relative potencies capture different aspects of $B_{c_{i}}$ , they lead to different optimal solutions. However, selective compound-target pairs identified using both statistics can be considered together, based on the needs of the user.

### 2.3 The integrated target-specific compound selectivity score

When applying the bi-objective optimization to identify selective compound-target pairs, an integrated selectivity score can be calculated by combining both the local and global relative potencies to quantify the selectivity of a compound for a given target. Such integrated selectivity score $S_{c_{i},\;t_{j}}$ for the compound-target pair (*c*_*i*_, *t*_*j*_) is formally defined as:

$${S_{c_{i},t_{j}}=\alpha ∙L}_{c_{i},t_{j}}+\left(1-\alpha \right)∙G_{c_{i},t_{j}}$$ (3)

where the parameter α adjusts for the contributions of the local and global relative potency to the selectivity score.

The global relative potency $G_{c_{i},\;t_{j}}$ focuses on comparing the compound’s interaction strength against a specific target, relative to the average affinity to the other targets, and it therefore reflects the general interaction strength over $B_{c_{i}}\backslash \left\{K_{c_{i},\;t_{j}}\right\}$ . Large $G_{c_{i},\;t_{j}}$ indicates that the $K_{c_{i},\;t_{j}}$ is generally high compared to the $mean\left(B_{c_{i}}\backslash \left\{K_{c_{i},\;t_{j}}\right\}\right),$ but we note that (*c*_*i*_, *t*_*j*_) is not necessarily the only pair with strong interaction. For example, its nearest neighbor in terms of target potency, $K_{c_{i},\;t_{a}}$ , may be as high as $K_{c_{i},\;t_{j}}$ , meaning that compound *c*_*i*_ has similar interaction strength against *t*_*j*_ and *t*_*a*_. Therefore, the local relative potency $L_{c_{i},\;t_{j}}$ was introduced to better distinguish between $K_{c_{i},\;t_{a}}$ and $K_{c_{i},\;t_{j}}$ , since it emphasizes the local potency, relative to the average of neighbor targets, instead of all the other protein targets.

**FIGURE 4:**
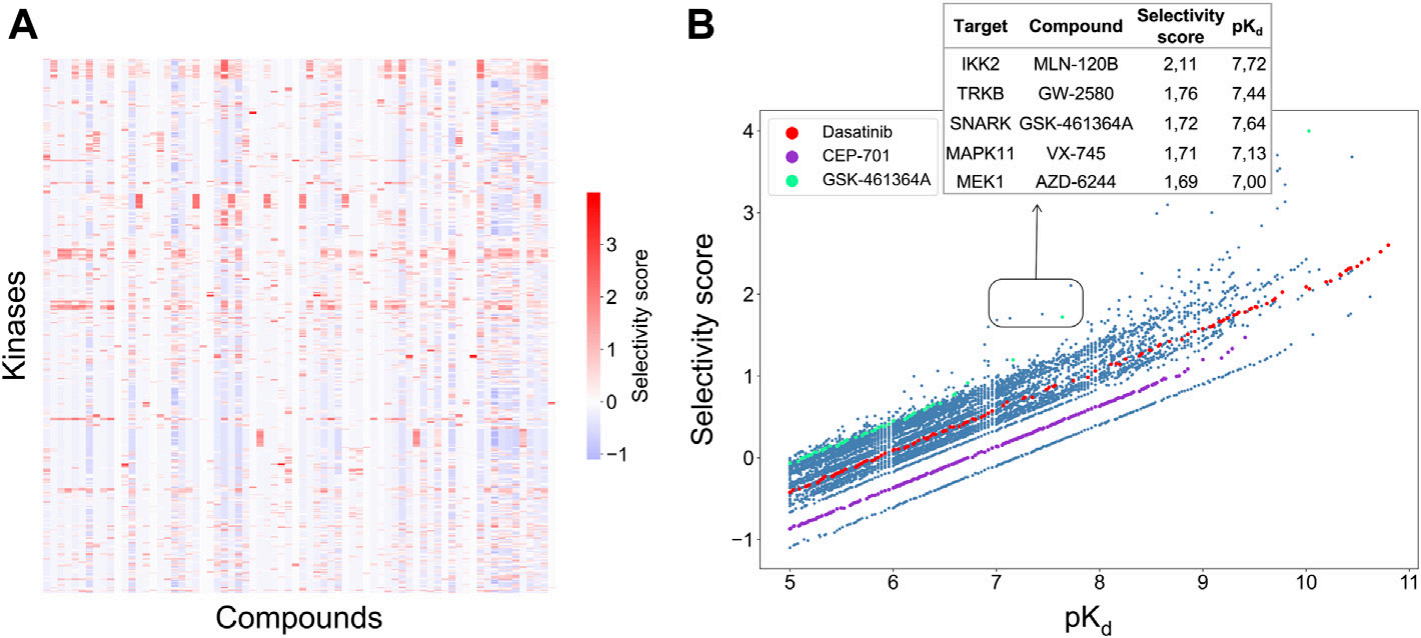
**(A)** Heatmap of the selectivity scores between 72 compounds and 442 kinases when using the weighting factor α = 0.5 in Eq. 3; **(B)** Correlation between the selectivity scores and absolute potency pK_d_ across the 31,824 compound-kinase pairs in the Davis dataset. Higher scores indicate higher selectivity. Examples of compound-kinase pairs with relatively low interaction strengths and high selectivity scores are highlighted in the box, and details shown in the inset table.

A weighted sum of the two relative potencies can be used to quantify the integrated selectivity of a compound-target pair to be optimized in Eq. 3. The weight of each relative potency term can be freely adjusted by the user. When more weight is placed on the local relative potency, then the selectivity score will focus more on distinguishing between the given target and its nearest neighbors in terms of the interaction strength, hence identifying compounds most potent against the given target in the context of the target neighborhood. As a default option, the mean of local and global relative potencies can be used (i.e., α = 0.5), if none of the terms is considered more important than the other in the particular drug discovery or repurposing application (Figure 4A).

Figure 4B shows the correlation between the integrated selectivity scores and pK_d_ values, colored for three example compounds discussed below. In general, and as was expected, a higher interaction strength (absolute potency measured by pK_d_) corresponds to higher selectivity. However, by combining the local and global relative potencies, one can discover compounds that are selective, yet may have relatively weak interaction strengths. For example, MLN-120B has a relatively weak absolute potency of pK_d_ = 7.72 with IKK2, but it was identified as selective against IKK2 with a relatively high selectivity score of 2.11. In the Davis dataset, MLN-120B is the second most potent inhibitor of kinase IKK2, yet having the highest local relative potency. This example shows that with the adjustable weights, it is possible to reach a balance between compound potency and selectivity, with the aim to find maximally selective and potent compounds for a particular target of interest.

### 2.4 The application of target-specific selectivity score to kinase inhibition

**FIGURE 5:**
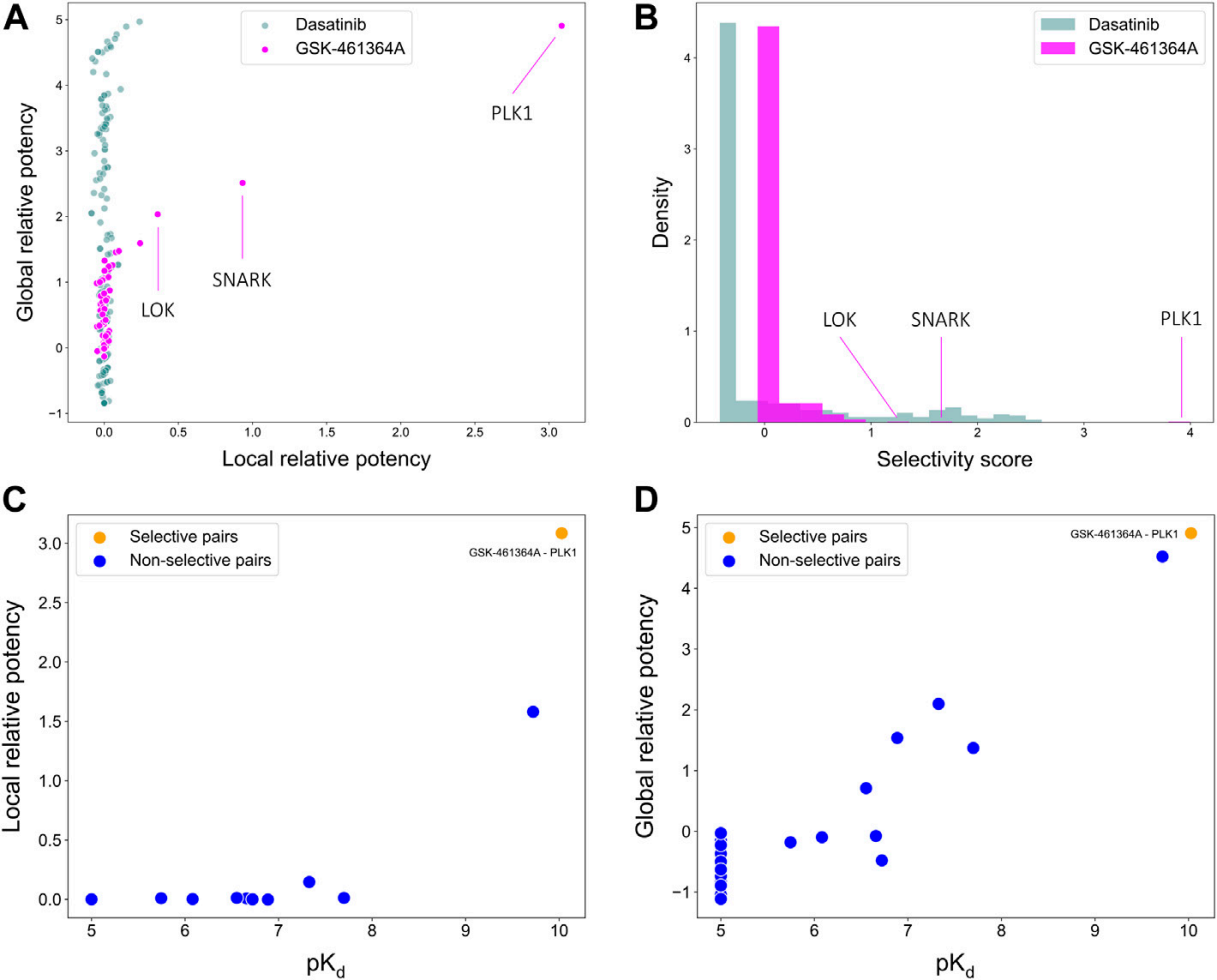
Comparisons of **(A)** local and global relative potencies and **(B)** distributions of selectivity scores for dasatinib and GSK-461364A across 442 kinase targets. GSK-461364A was identified through bi-objective optimization as selective against PLK1 using both **(C)** local relative potency and **(D)** global relative potency.

To illustrate the use of the target-specific selectivity score, Figure 5 shows the selectivity scores and relative potencies of two compounds: dasatinib and GSK-461364A. GSK-461364A is known to be highly selective against only a few kinase targets, PLK1, SNARK, and LOK, with much higher selectivity scores than for other kinases (Figure 5B). In contrast, dasatinib is a broad-spectrum multi-kinase inhibitor, and therefore many of its targets have high global relative potencies, but none of these targets have a high local relative potency (Figure 5A). A high global relative potency indicates that the compound shows overall selectivity to any target in general, since it considers the mean of $B_{c_{i}}\backslash \left\{K_{c_{i},\;t_{j}}\right\}$ . Thus, when considering dasatinib to be selective against a set of targets, more weight can be placed on the global relative potency; when searching for selective compounds against a few specific targets, more weight can be placed on the local relative potency. In this way, the target-specific compound selectivity score provides flexibility and becomes applicable to different drug discovery needs.

As shown above, GSK-461364A was identified as a highly selective PLK1 inhibitor since it has both high local and global relative potencies against PLK1 (Figures 5C,D). The bi-objective optimization also identified GSK-461364A as an optimally selective compound against SNARK and LOK, due to its high local relative potency (Supplementary Figure S1). For many kinase targets, such as PLK1, multiple highly selective compounds can be rather easily identified from the Davis dataset, but for some other targets, such as SNARK, LOK, and other targets shown in Supplementary Figure S1, compromises between the potency and selectivity need to be made through the bi-objective optimization. A *Pareto front* was generated to illustrate all the equally optimal compounds for a given target. For example, multiple compounds were identified as optimally selective for the kinase TNIK (see Supplementary Figure S1). The most selective compounds for the target can then be identified using the selectivity score (Eq. 3), along with other available information, including physicochemical properties of the compounds or their toxicity profile. In this way, the pareto optimization provides the user with additional quantitative information for the drug discovery process.

### 2.5 Evaluation of the stability of the target-specific selectivity score

**FIGURE 6:**
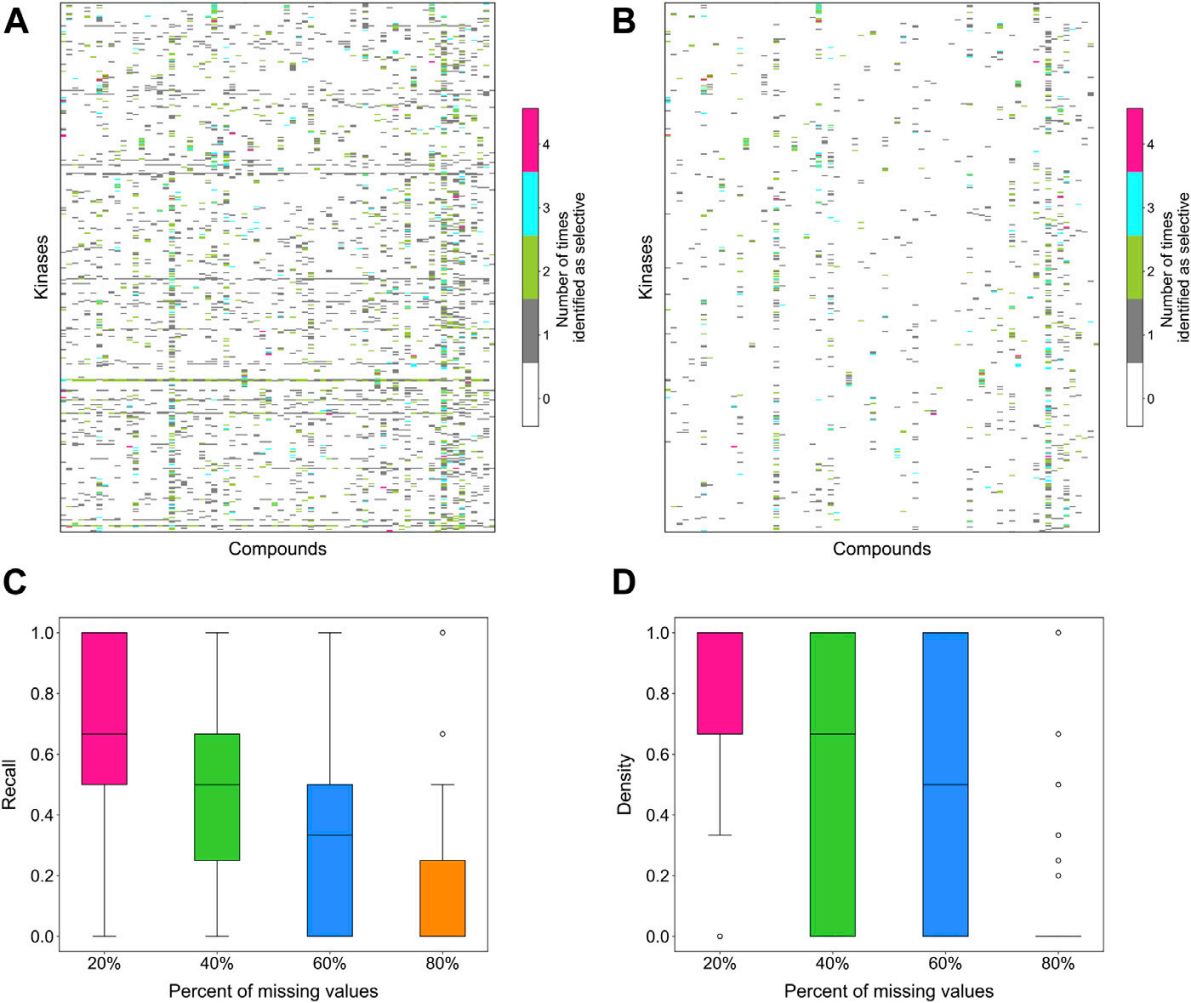
The number of times a compound was identified as selective for a target in bioactivity matrices with missing values when using **(A)** local relative potency and **(B)** global relative potency. Upper row: the heatmaps show the overall results in the matrix between 72 compounds and 442 kinases when adding 20, 40, 60 and 80% of missing values to the full bioactivity data matrix. In panel a, gray stripes correspond to kinases for which almost all compounds are identified as selective, indicating instability; Bottom row: the boxplots of the recall of identification of selective drug-kinase pairs from data matrices with missing values when using **(C)** local relative potency and **(D)** global relative potency, using the selective pairs identified in the full data matrix as ground truth.

To evaluate the stability of the target-specific selectivity score, we first studied the impact of missing bioactivity values by adding 20, 40, 60 and 80% of missing values to the full bioactivity data matrix, while keeping all the compounds and targets in the matrix. When considering all compound-target pairs, the global relative potency was in general more robust to missing data than the local relative potency (Figures 6A,B). For each kinase target, the recall value was calculated using the identified selective compounds from the full data matrix as true positives, using both local and global relative potencies (Figures 6C,D). As expected, the recall tends to decrease when increasing the missing value rates in the bioactivity data matrix. When only 20% of non-missing data are available, the recall values were distributed mostly at zero, suggesting that the identified selective pairs are not stable anymore. Based on the above results, the methodology appears reasonably consistent in bioactivity data matrices that have maximally 20% of bioactivity pairs missing.

Next, we studied the effects of various bioactivity data matrix sizes on the stability of the identifications. Data matrices of increasing sizes were subsampled from the full data matrix, with 20, 40, 60 and 80% of compounds and targets included, and the selective compound-target pairs were identified based on each subsampled matrix. For a compound-target pair, the number of times it was identified as selective in the submatrices of different sizes was considered as a measure of consistency. If a compound-target pair was identified as selective in all the data matrices, regardless of the bioactivity matrix size, it indicates that even with a very small data size, for example 20% of the compounds and targets that corresponds to 4% of the full data matrix, the method can still identify the selective pairs, and the result is consistent with that when using the larger bioactivity data matrices.

Supplementary Figure S2 shows the overall heat map counting the occurrences of selective pairs consistently identified across different sizes of submatrices. A count of 5 means a compound-target pair was identified as selective in all submatrices of different sizes, and a count of 1 means a compound-target pair was identified only once as selective. Some compound-target pairs were only present in the largest data matrix, i.e., the full data matrix, thus they can only be identified once. Similar to Figure 6, the selectivity score tends to be more stable when using global relative potency, as the identified pairs are more consistent compared to that when using the local relative potency (Supplementary Figure S2A). As expected, gradually decreasing the data matrix size leads to identification of certain targets with many selective compounds, indicating increased instability. In general, when the data size is the smallest, i.e., 4% of the full data matrix, the method starts to behave inconsistently, suggesting that larger data matrices are required.

### 2.6 Statistical evaluation of the relative potency using empirical *p*-values

**FIGURE 7:**
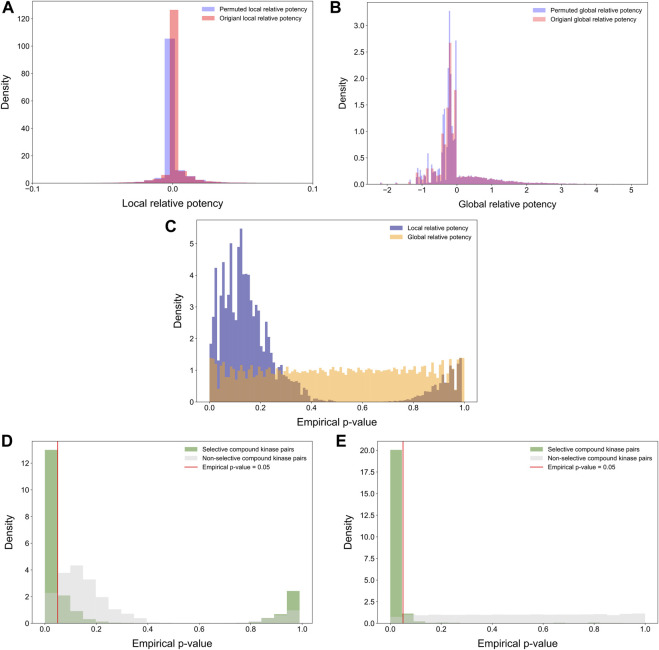
Distributions of **(A)** permuted and original local relative potencies; and **(B)** permuted and original global relative potencies; **(C)** the empirical *p*-values calculated with permutation procedure for both local and global relative potency; empirical *p*-values of **(D)** local relative potency and **(E)** global relative potency colored by whether the compound-kinase pair is identified as selective or not.

Statistical properties of the relative potency were next studied by randomly permuting the compound-target bioactivity matrix. Local and global relative potencies were calculated based on the permuted matrices to form the background distribution for null hypothesis. As expected, the background distributions were concentrated at around zero, especially for the local relative potency (Figures 7A,B). Next, the empirical *p*-values were calculated for each compound-target pair based on the background distributions (Figure 7C). The empirical *p*-values for the global relative potency were almost uniformly distributed, as would be expected for a proper statistic, but for the local relative potency, the *p*-values tend to be either very small or close to 1. The ill-distributed *p*-values of local relative potency may be due to the local neighborhood size (*h* = 5) that was used as default in its calculation. When comparing the *p*-value distributions of compound-target pairs identified as selective with those of non-selective pairs, it was observed that *p*-values for selective pairs are more concentrated around zero, i.e., indicating statistically significant target-specific selectivity (Figures 7D,E).

We note that the local relative potency is closely related to the global relative potency, since when the number of neighbors *h* is increased to all the targets, the local relative potency becomes equal to the global relative potency. Thus, we wanted to study the effect of using increasing numbers of nearest neighbors when calculating the local relative potency for the bi-objective optimization. In general, different numbers of nearest neighbors resulted in rather similar detections, which are distinct compared to using the global relative potency (Supplementary Figure S3A). When comparing the identified selective compounds per target, using the local relative potency based on different numbers of nearest neighbors, we calculated recall values using selective compounds identified by the global relative potency as true positives. The recall distributions showed that the performance of the local relative potency is again relatively consistent when the number of nearest neighbors varies (Supplementary Figure S3B). Taken together, the consistent behavior of the local relative potency calculation indicates that the two versions of the relative potency capture both unique and common properties of the compound-target interactions.

### 2.7 Comparison of target-specific selectivity with existing selectivity metrics

Since most of the existing compound selectivity metrics are designed only from the perspective of compound selectivity, it is not straightforward to make comparisons between those metrics and our target-specific selectivity metric. Furthermore, the metrics are also designed for different bioactivity readouts, and may have different directions and scales to indicate selectivity. To make a reasonable comparison, we z-scaled and standardized all the metrics so that the smaller the metric, the more selective the compound (see Materials and Methods). For our target-specific selectivity score, we used a summarized, target-agnostic selectivity score, calculated as the mean of selectivity scores of a compound across all available targets. We also used the number of identified selective targets for each compound as a measure of the compound’s overall selectivity, regardless of the target. Supplementary Figure S4 shows that such summarized measures coincide among the selectivity metrics, since in effect, they all measure whether a compound has a strong activity against multiple or only a few targets. For example, the local and global relative potencies correlated well with the standard score using pK_d_ of 7 as the activity cut-off (Supplementary Figure S4 **)**.

**FIGURE 8:**
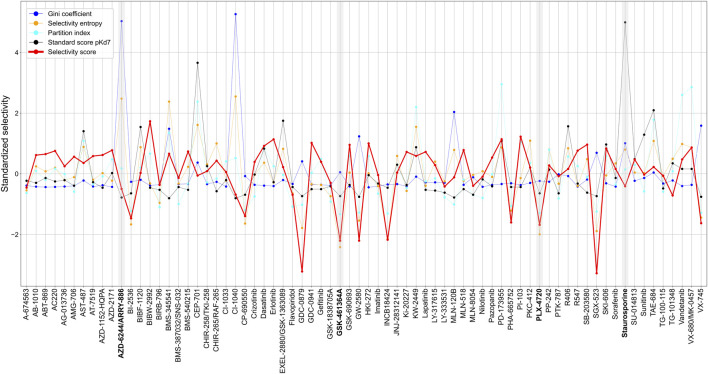
Comparison of various target-agnostic compound selectivity metrics. Local and global relative potencies are summarized along the targets of each compound in the target-specific selectivity metric, and all the metrics are z-scaled and standardized so that the smaller metric values indicate more selective compounds. The boldfaced compounds are discussed in the text.

Across the 72 kinase inhibitors in the Davis dataset, most of the target-agnostic summary metrics identified selective and broadly-active compounds rather consistently, expect for the Gini coefficient that was not highly correlated with the other metrics (Figure 8). This could be due to the different data types required by Gini coefficient, which was designed for percent inhibition values instead of K_d_ data. For example, dasatinib and staurosporine are two well-known broadly-active kinase inhibitors, and they were considered as non-selective by most of the metrics. Similarly, more target-specific compounds, such as GSK-461364A and PLX-4720, were identified as highly selective compounds by most of the summary metrics. As an exception, AZD-6244 was considered non-selective in terms of Gini coefficient and selectivity entropy, with relatively high scores compared to other compounds in the dataset, whereas AZD-6244 was considered relatively selective by our selectivity score and the standard score. Upon inspecting the Davis dataset, AZD-6244 has interaction strengths of pK_d_ >5 with 13 out of 442 kinases, which are mainly MEKs and EGFR mutants (Supplementary Figure S5).

These results demonstrate a consistent performance of our target-specific selectivity metric, when using it to measure the overall target-agnostic compound selectivity.

### 2.8 Comparison of target-specific selectivity with partition index

**FIGURE 9:**
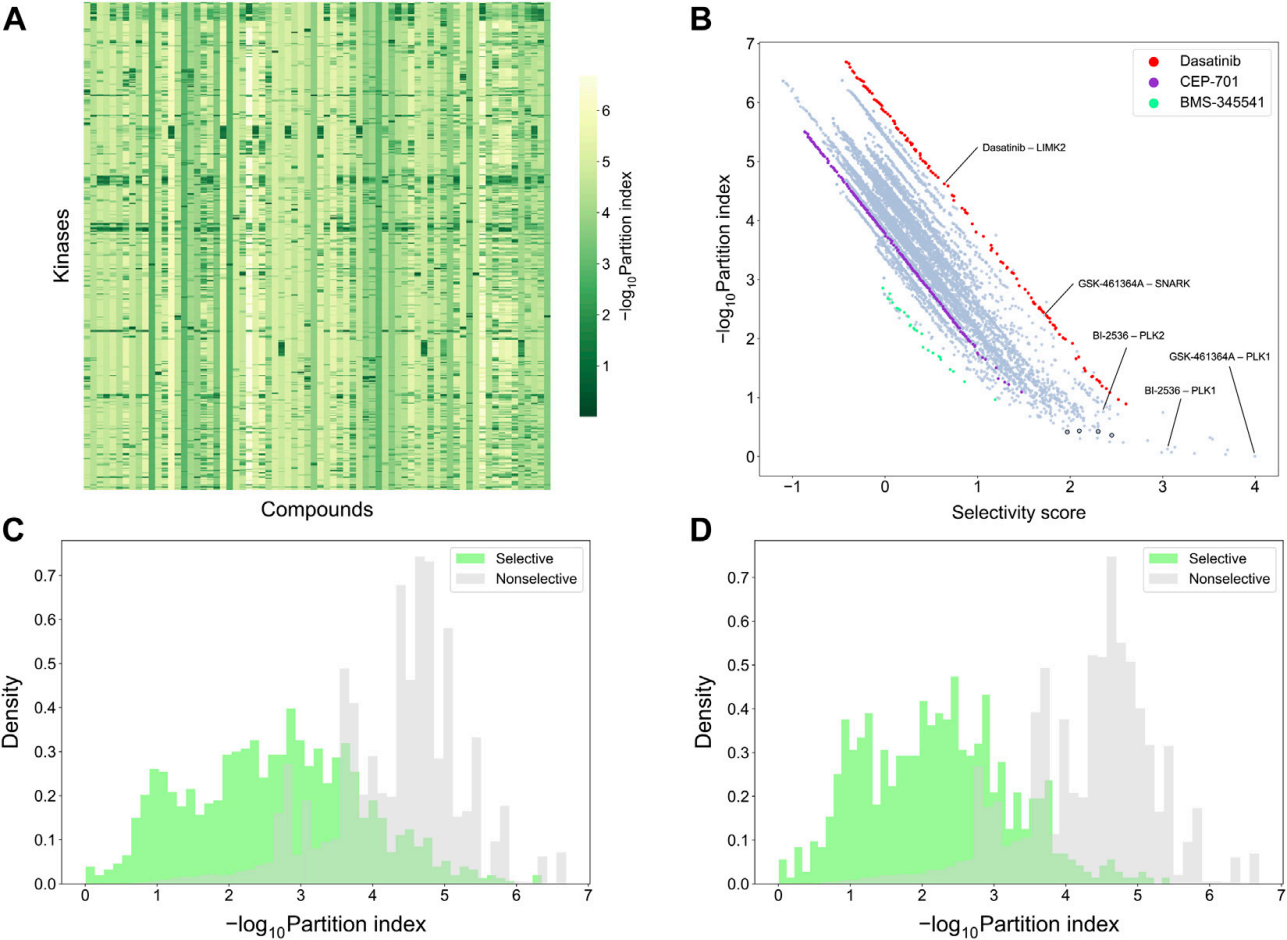
Comparison of target-specific selectivity score and partition index. Upper row: **(A)** Heatmap of -log10 (partition index) for each kinase, where smaller values indicate more selectivity; **(B)** correlation between partition index and selectivity score across the 31,824 compound-kinase pairs in the Davis dataset; Bottom row: distributions of -log10 (partition index) colored by whether the compound-kinase pair was selective when using **(C)** local relative potency and **(D)** global relative potency.

To make a more detailed, target-specific comparison, the partition index scores were calculated such that each kinase target was used separately as the reference target (see Figure 9A which shows the negative logarithm of the target-specific partition indices). The vertical stripes indicate that the partition index considers many compounds to be selective against all the targets, suggesting that the partition index is not generally capable of finding selective compound-target pairs. When comparing the partition index with our target-specific selectivity score, it was observed that the two metrics are generally well correlated, as expected, but the new selectivity metric was more distinctive in terms of identifying selective compound-target pairs (Figure 9B). Especially, when the partition index is small, between 0 and 1, the selectivity score can still distinguish between the highly selective and broadly-active kinase inhibitors better than the partition index.

Supplementary Table S1 shows several example compound-target pairs that have low partition indices, yet higher and more different target-specific selectivity scores (the black bordered points in the bottom right corner of Figure 9B). For example, the compound-target pairs (nilotinib, DDR1) and (PTK-787, KIT) have selectivity scores of 2.30 (0.11% quantile) and 1.97 (0.38%), respectively, while their partition indices are 0.43 (0.057%) and 0.42 (0.053%), suggesting that target-specific selectivity score provides slightly better separation for the pairs, as further supported by the significant *p*-values, using both local and global relative potency (Supplementary Table S1). Such observations suggest that the new selectivity score harnesses different information than the partition index, thus providing additional perspective to the target-specific discovery or repurposing of selective compounds.

To further compare the two selectivity approaches, Figures 9C,D shows the distributions of the partition index for the compound-target pairs identified as selective or non-selective by the target-specific selectivity score. Regardless of whether using the local or global relative potency, the partition indices of the selective pairs tend to have lower values than those of non-selective pairs, indicating that the two methods are generally consistent with each other. However there exists also pairs identified as selective by the target-specific score, yet having a relatively large partition index values, or vice versa, shown as the overlaps of two distributions in Figure 9C,D. For example, the pair (BI-2536, PLK1) has a very low partition index of 0.13, indicating relatively high selectivity. In the Davis dataset, GSK-461364A is the most potent inhibitor of PKL1 (pK_d_ = 10.03), with BI-2536 being the second most potent (pK_d_ = 9.72) (Supplementary Figure S5). From the compound perspective, both compounds have their highest potency against PLK1. However, GSK-461364A has a pK_d_ of 7.64 for its second most potent target (SNARK), while BI-2536 has a pK_d_ of 9.09 against PLK2. Since BI-2536 has very similar potencies against its top-2 most potent targets, it is not considered as selective against PLK1 when GSK-461364A is available in the library. These examples further demonstrated that our method provides an added value for finding target-specific selective compounds.

## 3 Discussion

Finding selective compounds is considered important for kinase drug discovery since many of the current kinase inhibitors are relatively promiscuous. This is the case also with many kinase inhibitors marketed or under current development (Cohen et al., 2021), and it remains a challenging task to find more targeted and selective inhibitors that can both improve efficacy and reduce the unwanted off-target toxicity (Attwood et al., 2021). Our results show that the new target-specific selectivity score provides an added value for the discovery of multi-targeting, yet selective compounds in the case when a target of interest is pre-defined. The selectivity score derived from the relative potencies measures the target-specific compound selectivity quantitatively and provides flexibility for the user. The bi-objective optimization was capable of identifying the maximally selective compound-target pairs in the presence of a wide degree of polypharmacological effects. The flexibility comes from the user-adjustable weights for the local and global relative potencies in the selectivity score, as well as from using both the relative and absolute potency in the bi-objective optimization. Such flexibility allows wide applications, based on different user needs, for example, finding the most selective compound for a single target or group of targets. Thus, the new metric is expected to become beneficial in kinase inhibitor development, and more broadly in lead compound identification in drug discovery and for repurposing multi-targeting drugs.

The advantage of the target-specific selectivity score is that it requires only the bioactivity measurements of the compound-target pairs, without the need to provide other information of the compounds, such as their on/off targets or chemical structures. This makes our approach widely applicable to various types of bioactivity measurements. In case the available bioactivity data contains various studies of target activities using multi-dose assays, such as a mix of K_i_, K_d_ and IC_50_ readouts, then the bioactivity readouts can be summarized and integrated using our previously developed data transformations (Wang et al., 2020). Due to its data-driven approach, the approach is not only limited to kinase inhibitors, but once sufficient amounts of similar bioactivity data become available for other target classes, such as G-protein-coupled receptors (GPCRs), the same approach is directly applicable to these data. Apart from calculating the target-specific selectivity score, the approach also provides optimal solutions of the most selective compound-target pairs based on the given bioactivity data. Finally, the target-specific selectivity enables the user to find selective compounds for the particular targets of interest. Such target-specificity provides a unique perspective to analyzing compound selectivity, and expands the application area of the current compound selectivity metrics in multi-target drug discovery and repurposing.

The limitation of any data-driven approach is the data availability and quality. Since the target-specific selectivity approach requires experimentally measured bioactivity data, we recommend that at least 80% of the compound-target pairs should have measured bioactivities to obtain a reliable performance. Such a requirement limits the approach to only compounds with sufficient amounts of target bioactivity measurements available. However, the approach can be further developed by incorporating other information of either compounds or targets, for example, compound structural similarity (Lo et al., 2019) to infer selectivity of novel compounds, even without any measured bioactivities. Alternatively, machine learning methods can be used to predict bioactivities for the compound-target pairs that have not yet been explored experimentally (Bora et al., 2016; Merget et al., 2017; Öztürk et al., 2018; Thafar et al., 2019; Vamathevan et al., 2019; Bagherian et al., 2020; Nguyen et al., 2020; Schneider et al., 2020; Cichońska et al., 2021; Ye et al., 2021), after which the target-specific compound selectivity metric can be applied to the fully predicted compound target interaction matrix to identify selective lead compounds against any target of interest. In the general method development, we did not distinguish between the on- and off-targets, or penalized targets that may lead to adverse effects in clinical applications, but such factors could be later incorporated into the general selectivity scoring approach when applied to a particular disease or cellular context, similar to the KInhibition Selectivity Score (Bello and Gujral, 2018), but this will require careful distinction between the therapeutic and toxicity-related targets.

## 4 Conclusion

We have developed a novel target-specific compound selectivity metric by decomposing the selectivity into absolute and relative potencies. Two statistics were used to describe the relative potency, local and global relative potencies, which characterized the target-specific compound selectivity from different aspects and can be combined using a weighted sum as the integrated selectivity score to facilitate the quantification of compound selectivity. A bi-objective optimization problem was used for maximizing both absolute and relative potencies to identify the maximally target-specific selective compounds in a given compound-target interaction dataset. The new selectivity approach is expected to contribute to finding selective compounds with improved target-specificity, as well as to enable repurposing of existing multi-targeting drugs for new disease indications that are driven by the specific disease protein.

## 5 Materials and methods

The workflow of the target-specific compound selectivity scoring is illustrated in Supplementary Figure S6.

### 5.1 Compound-target interaction data for method development

The compound target activity data used to develop and test the target-specific compound selectivity were obtained from Davis et al. (Davis et al., 2011), hereby called the Davis dataset. In the Davis dataset, dissociation constant K_d_ was measured for all pairs between 72 compounds and 442 kinases. In our analyses, pK_d_ = -log_10_(K_d_) is used, and the larger is the pK_d_ the stronger the binding affinity.

### 5.2 Decomposition of target-specific compound selectivity

Similar to our previous work on identification of selective drug combination treatment effects (Pulkkinen et al., 2021), two aspects of compound binding properties were considered to quantify target-specific selectivity (1): the compound’s potency against the target of interest, termed the *absolute potency*; and (2) the compound’s potencies against other targets, termed the *relative potency*. To find a selective compound for a given target protein, we consider that the compound needs to be potent enough against the target, and simultaneously, it must have a weak or no activity against the other potential targets.

The absolute potency can be basically any multi-dose bioactivity measurement, such as K_i_, K_d_, IC_50_ or EC_50_, which measures the binding affinity between the compound and target of interest. The relative potency can be quantified in different ways, for example, as the difference between the absolute potency and the mean of a compound’s potencies against all the other targets, except for the target of interest. Such relative potency uses as reference the compound’s overall binding affinity with all other targets, thus termed as *global relative potency*. A more focused measure of relative potency is to consider only those targets having the closest potencies to the target of interest, for example, the difference between the absolute potency and the mean of *h* nearest neighbors’ potencies with the target of interest. Such calculation measures the compound’s average interaction strength within the local neighborhood of the target of interest, thus termed as *local relative potency*. If the mean value is higher than the absolute potency, this indicates that the compound has similar or stronger binding activity with several targets.

### 5.3 Bi-objective optimization to identify target-specific selective compounds

Selectivity score provides a quantitative tool to understand and quantify target-specific compound selectivity. However, in most cases, it is difficult to find the optimally selective compound for a specific protein target. Therefore, we used bi-objective optimization to find the most selective compound-target pairs given a particular compound-target interaction dataset. Two separate bi-objective optimization problems were solved to identify target-specific selective compounds (1): maximizing both absolute potency $K_{c_{i},\;t_{j}}$ and local relative potency $L_{c_{i},\;t_{j}}$ (2); maximizing both absolute potency $K_{c_{i},\;t_{j}}$ and global relative potency $G_{c_{i},\;t_{j}}$ .

Let us denote by $K_{c_{i},\;t_{j}}$ the binding strength of a compound *c*_*i*_ from a set of compounds $C=\left\{c_{i}\right\}$ against a target protein *t*_*j*_ from a set of protein targets $T=\left\{t_{j}\right\}$ . The activity spectrum of a compound *c*_*i*_ can then be defined as $B_{c_{i}}=\left\{K_{c_{i},\;t_{j}}\;|{\;t}_{j}\in T\right\}$ .

For the optimization formulation, the two relative potencies are formally defined as follows:

Local relative potency:

$$L_{c_{i},t_{j}}=K_{c_{i},t_{j}}-\frac{1}{n}\sum_{h=1}^{n}K_{\mathrm{hNN}\left(c_{i},t_{j}\right)}$$

where $K_{{hNN(c}_{i},\;t_{j})}$ denotes the absolute potency of hth nearest neighbor of *t*_*j*_ given *c*_*i*_.

Global relative potency:

$$G_{c_{i},t_{j}}=K_{c_{i},t_{j}}-\frac{1}{\left|T\right|-1}\sum_{l=1}^{\left|T\right|}K_{c_{i},t_{l}}\;,\;\left(l\neq j\right)$$

The bi-optimization problem is to maximize both the absolute potency and the relative potency, which can be solved using the ε-constraint method (Haimes, 1971; Miettinen, 1999) as follows:

$$\underset{\varepsilon \in R}{⋃}\left\{\underset{c,\;t}{argmax}K_{c,\;t}|\;L_{c,\;t\;}<\varepsilon ,\;c\in C,\;t\in T\right\}$$

$$\underset{\varepsilon \in R}{⋃}\left\{\underset{c,\;t}{argmax}K_{c,\;t}|\;G_{c,\;t\;}<\varepsilon ,\;c\in C,\;t\in T\right\}$$

Here, the relative potency can be calculated either by local or global relative potency, $L_{c,\;t}$ or $G_{c,\;t}$ , respectively. We used *h* = 5 as default neighborhood size in the local relative potency.

### 5.4 Evaluation of target-specific selectivity

We carried out several analyses to evaluate the performance and stability of the target-specific selectivity score.

#### 5.4.1 The effect of matrix size and missing bioactivity values

We first studied the effect of compound-kinase interaction matrix sizes on the identification of selective compound-kinase pairs. Increasingly sized submatrices were sampled using 20, 40, 60, 80 and 100% of the compounds and kinases in the full matrix, respectively. In each submatrix, the same selectivity identification method was applied to generate a binary matrix with 0 indicating non-selective and 1 selective compound-kinase pairs. The matrices were aligned by the identity of compounds and kinases and added up accordingly. For example, all the five submatrices contain the first 20% of the compounds and kinases. Therefore, the sum of the binary matrices, which ranges between 1 and 5, indicates how well the method reproduces the same selectivity identification for the compound-kinase pairs present in the particular part of the matrix.

Next, the effect of missing bioactivity values was studied. For each kinase, 20, 40, 60, 80% compounds were randomly subsampled from the set of all compounds, and these were assigned as missing, to form matrices with random artificial missing values. Such matrices were generated with 20, 40, 60, 80% missing values independently (i.e., missing completely at random). The same selectivity identification method was applied to all the matrices. The identified selective compound-kinase pairs from each subsampled matrix were compared to those identified based on the original full data matrix, without missing data, to study the effect of increasing the amount of missing data.

#### 5.4.2 Permutation procedure to calculate empirical p-values

The original compound-kinase bioactivity matrix was randomly shuffled for 10,000 times, corresponding to a bioactivity matrix between compounds and kinases where the labels of the compounds/kinases were randomized, and the identification method was applied to each of those randomized matrices to form the background distributions for the local and global relative potencies. Then, for the observed local and global relative potencies calculated from the original matrix, empirical *p*-values were calculated as the percentage of values in the background distribution smaller or equal than the observed local and global relative potencies, respectively.

#### 5.4.3 Relationships between h, local and global relative potency

To study the effect of the number of nearest neighbors *h* used in the calculation of the local relative potency, an increasing number of 1, 5, 20, 100 nearest neighbors were used to calculate the local relative potency. Then, for each kinase, the number of identified selective compounds was compared among the local relative potencies when using different numbers of nearest neighbors.

The local relative potency becomes equal to global relative potency when setting the number of nearest neighbors equal to all available neighbors, i.e., *h* = |*T*| - 1. Therefore, selectivity identified using global relative potency was considered as the ground truth, against which the selectivity identified using different local relative potencies were compared, and the recall values were calculated:

$$Recall=\frac{TP}{P}$$

Here, *TP* is the number of true positives, i.e., the overlap between the selective compound-target pairs identified both by the local relative potency, using different numbers of nearest neighbors, and by the global relative potency, considered as the ground truth. *P* is the number of positive cases, i.e., the selective compound target pairs identified by the global relative potency.

### 5.5 Comparison of compound selectivity metrics

#### 5.5.1 General compound selectivity metric comparison

Since most of the existing compound selectivity metrics are not target-specific, we used the number of selective targets identified for each compound as a target-agnostic selectivity metric based on our target-specific selectivity approach to make a fair comparison with the other selectivity metrics. Different metrics may also have different ranges as well as different directions. Thus, for comparison, all the metrics were normalized to zero mean and unit standard deviation using the z-scaling:

$$z=\frac{x-\mu }{\sigma }$$

where *x* is the value of the original selectivity score, and *μ* and *σ* are the mean and standard deviation of the original selectivity scores, respectively. All the metrics were also normalized in direction, such that the smaller the value of the metrics, the more selective is the compound.

#### 5.5.2 Target-specific compound selectivity comparison

As described in the original work (Cheng et al., 2010), partition index can be considered as a target-specific compound selectivity metric when choosing specific reference target. Thus, we calculated partition index for each compound-target pair separately as follows:

$$Partition\;index\;of\;\left(c_{i},t_{j}\right)=\frac{\frac{1}{K_{c_{i},t_{j}}}}{\sum_{t_{j}}\frac{1}{K_{c_{i},t_{j}}}}$$

This calculation was then compared with our target-specific compound selectivity score calculated from the local and global relative potency. In negative logarithm form, the smaller the partition index, the more selective is the compound-target pair.

#### 5.6 Software tools

Python programming language (version 3.7, https://www.python.org) was used for all the analyses. Python libraries Pandas (version 1.3.4) (McKinney and W, 2010; Reback et al., 2020) and Numpy (version 1.21.2) (Harris et al., 2020) were used for data processing and bi-objective optimization. Python libraries Matplotlib (version 3.5.1) (Hunter, 2007), Seaborn (version 0.11.0) (Waskom, 2021) and venn (0.1.3, https://pypi.org/project/venn/) were used for making the figures.

## Data Availability

Publicly available datasets were analyzed in this study. This data can be found here: https://static-content.springer.com/esm/art%3A10.1038%2Fnbt.1990/MediaObjects/41587_2011_BFnbt1990_MOESM5_ESM.xls.
