# Supplementary material for: Target-specific compound selectivity for multi-target drug discovery and repurposing
Source: Front Pharmacol. 2022 Sep 23;13:1003480. doi: 10.3389/fphar.2022.1003480 (PMC9549418; doi:10.3389/fphar.2022.1003480)
Supplement: Supplementary file 1 [file DataSheet1.pdf]

Supplementary Material for

Target-specific compound selectivity for multi-target drug discovery and repurposing

Tianduanyi Wang[1,2], Otto Pulkkinen[1,3,4], Tero Aittokallio[1,3,4,5, 6]

[1]Institute for Molecular Medicine Finland (FIMM), University of Helsinki, Helsinki, Finland

[2]Department of Computer Science, Aalto University, Espoo, Finland

[3]Helsinki Institute for Information Technology (HIIT), Department of Computer Science, University of Helsinki, Helsinki, Finland

[4]Department of Mathematics and Statistics, and InFLAMES Research Flagship, University of Turku, Turku, Finland

[5]Institute for Cancer Research, Department of Cancer Genetics, Oslo University Hospital, Oslo, Norway

[6]Oslo Centre for Biostatistics and Epidemiology (OCBE), Faculty of Medicine, University of Oslo, Oslo, Norway

1 Supplementary Figures and Tables

1.1 Supplementary Figures

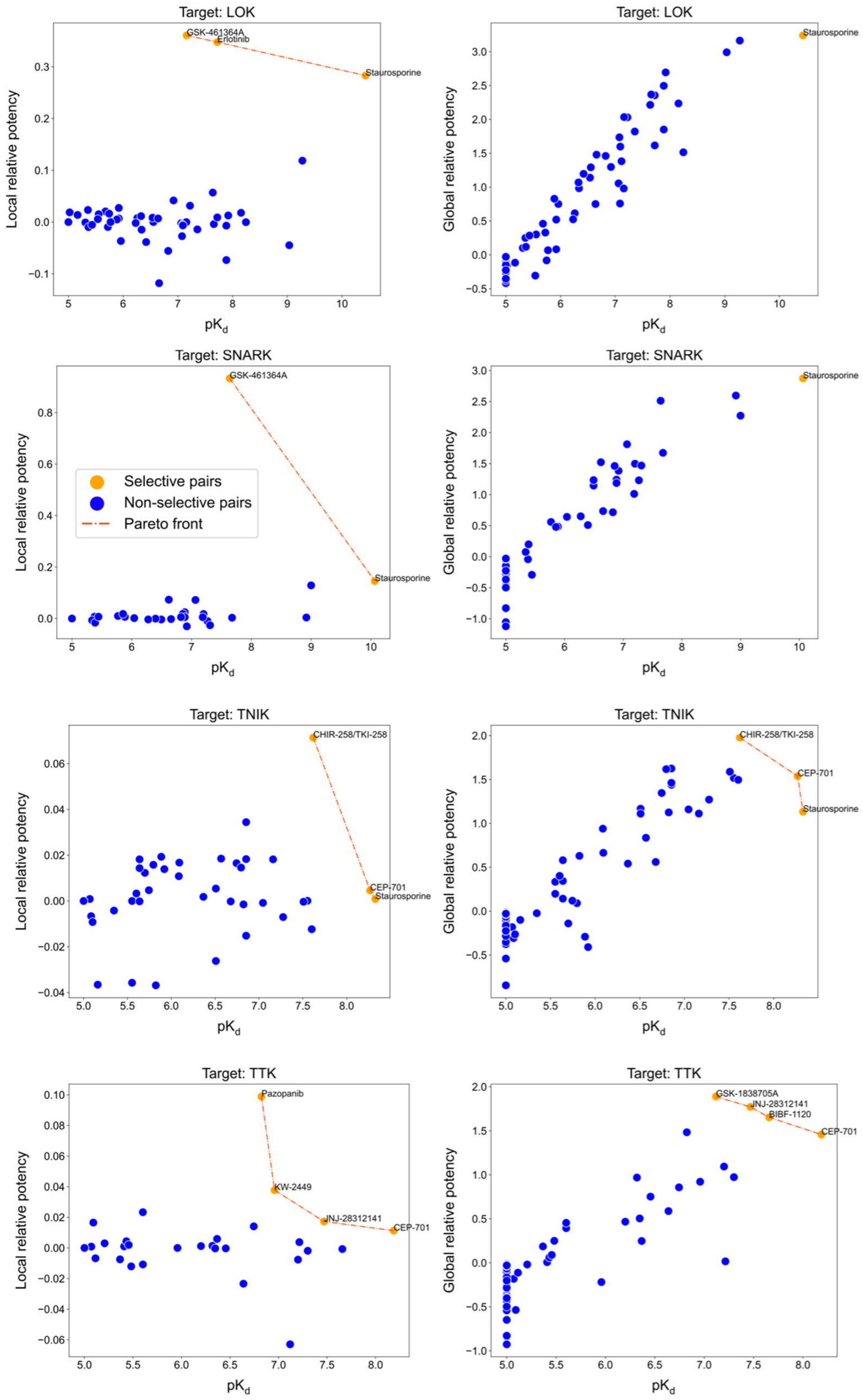

Fig. S1 Selective compound-target pairs identified as Pareto optimal solutions (Pareto front shown in orange) with the bi-objective optimizations using local (left) and global (right) relative potencies.

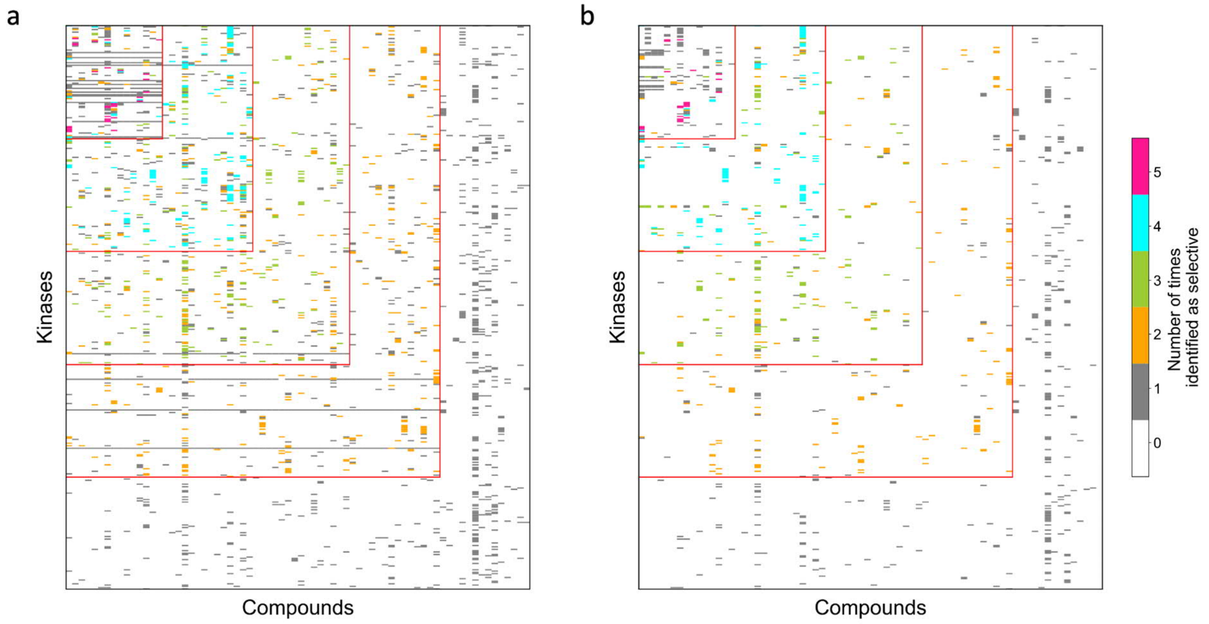

Fig. S2 The number of times a compound is identified as selective for a particular kinase target both in the subsampled and full matrices using (**a**) local relative potency and **(b)** global relative potency. The increasing submatrices contain 20%, 40%, 60% and 80% of the 72 compounds and 442 kinases from the full matrix. The horizontal stripes correspond to kinases for which almost all compounds are identified as selective, indicating instability at smaller data matrix sizes. The smallest sub-matrix contains 15 compounds and 89 kinases (4% of the full matrix). Higher numbers of compound-target pairs consistently identified as selective pairs across different sizes of submatrices indicate higher stability of the identification method.

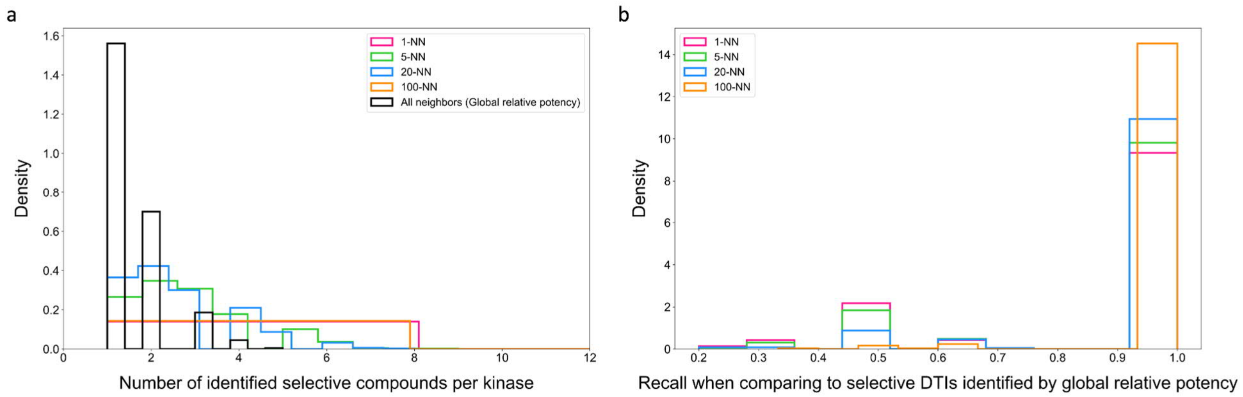

Fig. S3 Distributions of (**a**) the number of identified selective compounds per kinase; (**b**) the recall values using selective drug-kinase pairs identified using the global relative potency as true positives, when increasing the number of nearest neighbors in the local relative potency. When the number of neighbors is increased to all the targets, the local relative potency equals the global relative potency.

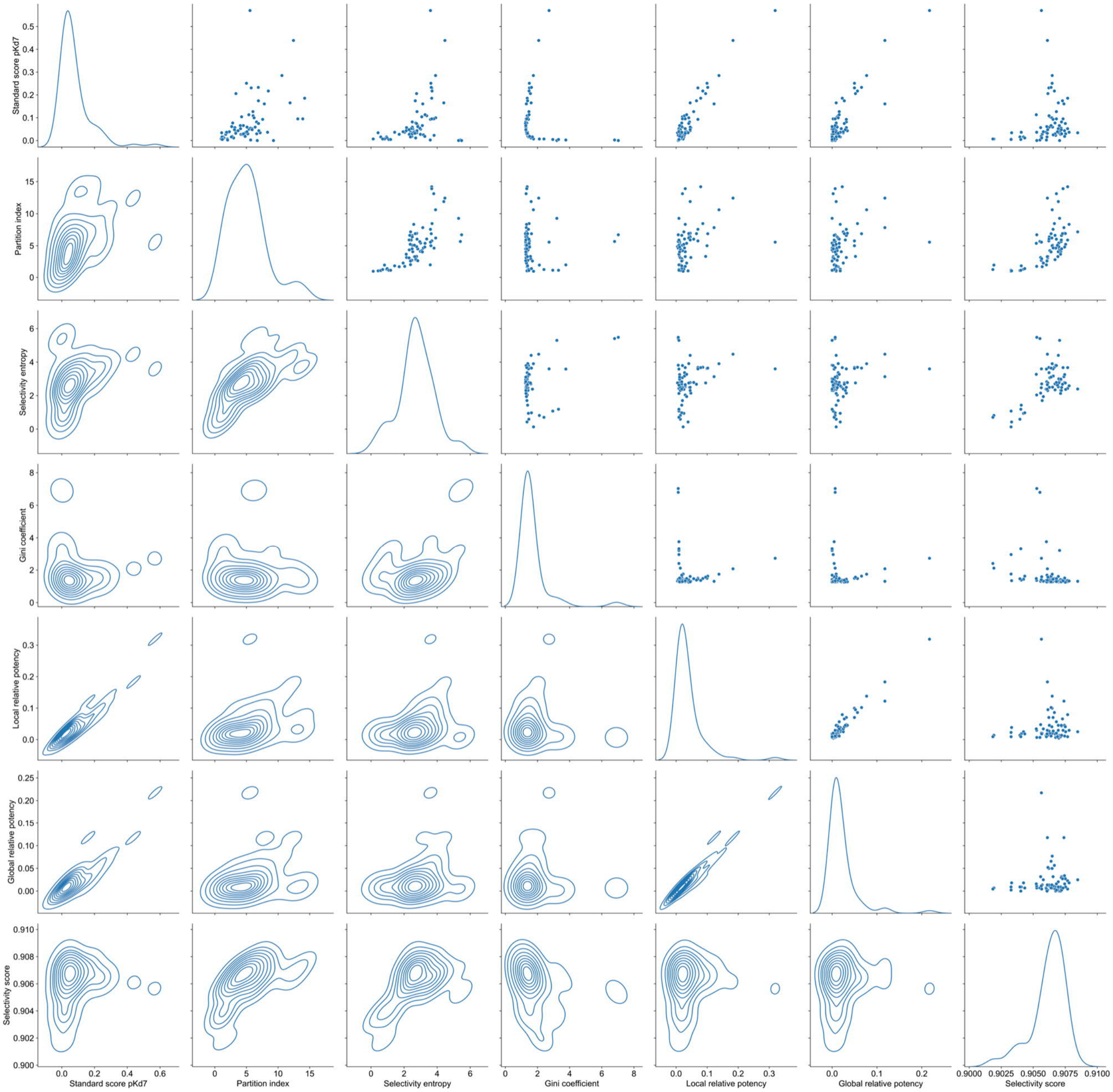

Fig. S4 Correlation coefficients and density distributions between different compound selectivity metrics. The local and global relative potencies are summarized along targets of each compound, and all the metrics are standardized so that the larger the metric the more selective is the compound.

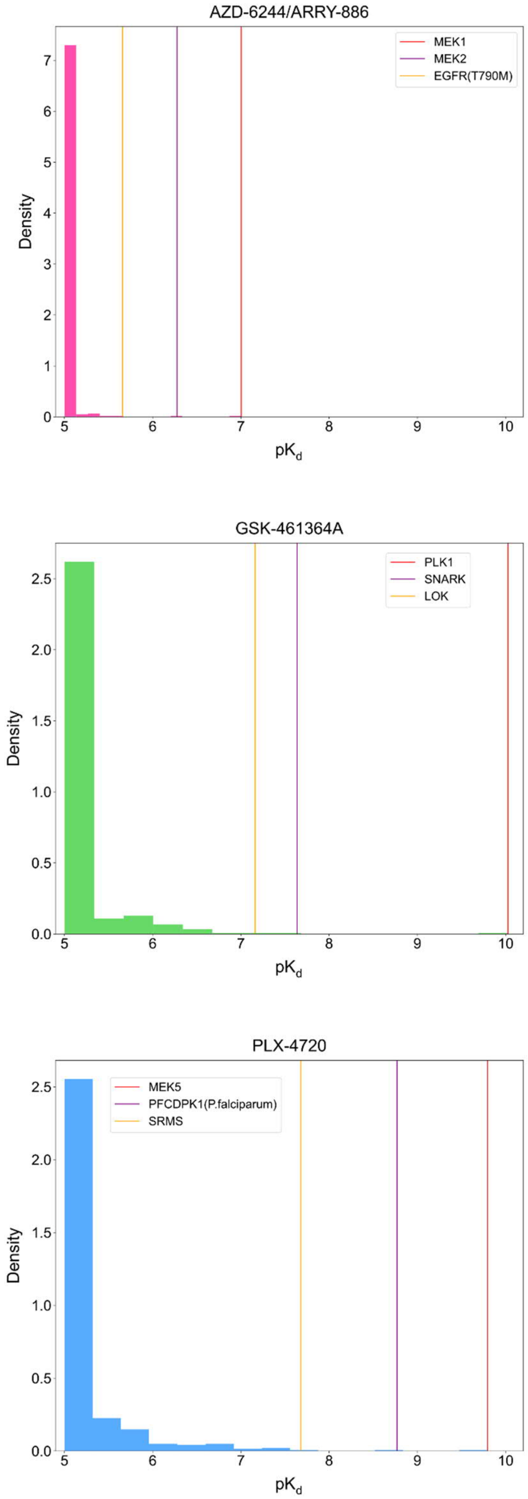

Fig. S5 Bioactivity pK_d distributions of three example compounds: AZD-6244/ARRY-886, GSK-461364A and PLX-4720; red lines indicate the most potent targets of the compounds, purple lines the second most potent, and orange lines the third most potent.

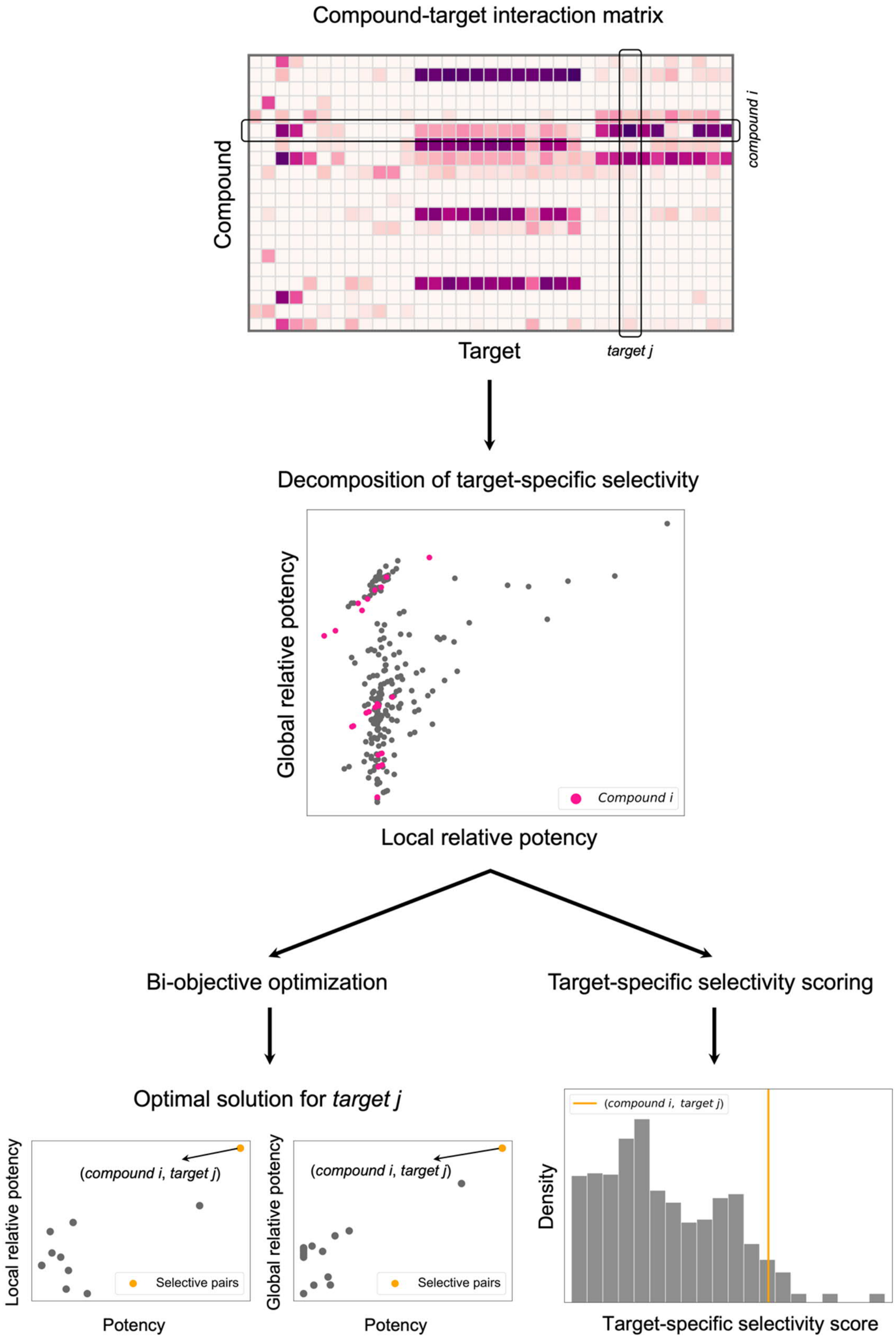

Fig. S6 Graphical illustration of the target-specific compound selectivity scoring workflow.

1.2 Supplementary Tables

Table S1. Examples of compound-target pairs identified in Figure 9b

Target	compound	Partition index	Selectivity score	pK_d	Local relative potency	Empirical p-value for local relative potency	Global relative potency	Empirical p-value for global relative potency
ALK	GSK-1838705A	0,36	2,45	9,26	0,86	0.0131	4,03	0.0001
KIT	PTK-787	0,42	1,97	8,29	0,70	0.0132	3,23	0.0967
DDR1	Nilotinib	0,43	2,30	8,96	0,92	0.0131	3,68	0.0151
PRKCQ	LY-333531	0,44	2,10	8,60	0,85	0.0138	3,35	0.0001
